# Influence of *Spirulina* and *Chlorella* Biomass on the Structure and Transport Properties of Electrospun PVA-Based Nanofiber Mats

**DOI:** 10.3390/nano16150929

**Published:** 2026-07-28

**Authors:** Margarita Neznakomova, Boris Mahltig, Mehmet Şen, Dilyana Gospodinova

**Affiliations:** 1 Faculty of Industrial Technology, Technical University of Sofia, 1000 Sofia, Bulgaria; m_neznakomova@abv.bg; 2 Faculty of Textile and Clothing Technology, University of Applied Sciences, 41065 Mönchengladbach, Germany; boris.mahltig@hs-niederrhein.de; 3 Department of Electrics and Energy, Technical Sciences Vocational School, Uludağ University, 16059 Bursa, Türkiye; mehmetsen@uludag.edu.tr; 4 Faculty of Electrical Engineering, Technical University of Sofia, 1000 Sofia, Bulgaria

**Keywords:** electrospinning, poly(vinyl alcohol), *Spirulina*, *Chlorella*, nanofibers, surface porosity, air permeability, water vapor permeability

## Abstract

Electrospun poly(vinyl alcohol) (PVA) nanofibrous mats containing *Spirulina* or *Chlorella* biomass were deposited directly onto a woven cotton fabric to produce fabric–nanomat composites. The effects of the microalgal species on the physicochemical properties of the electrospinning solutions, nanofiber morphology, nanomat development, surface porosity, and through-plane transport properties were comparatively investigated. The incorporation of either biomass increased the viscosity, electrical conductivity, and surface tension of the PVA solution, indicating interactions between the microalgal components and the polymer matrix. FTIR analysis showed that the nanomats largely retained the characteristic absorption bands of neat PVA, with minor spectral features consistent with the incorporated biomass. SEM observations revealed continuous, bead-free nanofibers without visible surface defects. The average fiber diameters were 192 ± 34 nm for PVA/*Spirulina* and 173 ± 39 nm for PVA/*Chlorella*. The PVA/*Chlorella* system produced thicker nanomats, whereas the PVA/*Spirulina* system exhibited greater projected-area expansion during prolonged electrospinning. Surface porosity was significantly higher for PVA/*Spirulina* nanomats (57.54 ± 1.34%) than for PVA/*Chlorella* nanomats (52.69 ± 1.49%). Correspondingly, the PVA/*Spirulina* composites exhibited higher air permeability, whereas both systems showed comparable relative water vapor permeability. These results demonstrate that the type of microalgal biomass influences nanomat development, morphology, porosity, and air permeability while having a limited effect on relative water vapor permeability, providing a basis for the development of functional textile and filtration-related materials.

## 1. Introduction

The development of sustainable materials derived from renewable resources has become an important research direction in response to growing environmental concerns associated with petroleum-based plastics [1]. Among the various biomass sources investigated, microalgae have attracted considerable attention due to their rapid growth rates, high photosynthetic efficiency, and ability to accumulate valuable biomolecules, including proteins, polysaccharides, lipids, pigments, vitamins, and antioxidants [1,2,3]. In addition to their sustainable production potential, microalgae possess a diverse chemical composition that enables their utilization in bioplastics, composites, biomedical materials, and other value-added applications [2,3]. Therefore, microalgal biomass is increasingly recognized as a promising raw material for the development of environmentally friendly functional materials [1,2,3].

Among the numerous commercially cultivated microalgae, *Spirulina* (*Arthrospira platensis*) and *Chlorella vulgaris* are among the most extensively studied species due to their rich biochemical composition and broad availability [2,4,5]. Although both microalgae are characterized by high protein content and the presence of biologically active compounds, significant differences exist in their cellular structure and chemical composition [2,4,6]. *Spirulina* is a filamentous cyanobacterium containing high levels of proteins, phycocyanin, essential fatty acids and minerals, whereas *Chlorella* is a unicellular green microalga rich in chlorophyll, carotenoids, vitamins and omega-3 fatty acids [2,4,5,6,7,8]. Furthermore, *Spirulina* exhibits a filamentous morphology, whereas *Chlorella* consists of nearly spherical unicellular particles enclosed by a rigid cellulose-rich cell wall [2,6]. These differences in morphology, cellular structure, and biochemical composition are expected to influence particle dispersion within the polymer matrix, polymer–filler interactions, and the electrospinning process, thereby affecting nanofiber formation and the structural and transport properties of the resulting composite materials [2,4,5,6,7,8].

To exploit the functional potential of microalgal biomass in nanostructured materials, an appropriate polymer matrix is required. Poly(vinyl alcohol) (PVA) is one of the most widely used water-soluble polymers owing to its excellent film-forming ability, biocompatibility, non-toxicity, and chemical stability [9,10,11]. In addition, the abundance of hydroxyl groups along the PVA backbone promotes hydrogen-bond formation with natural compounds and biomass-derived additives [9,10,11]. These interactions facilitate the incorporation of microalgal particles into the PVA polymer matrix and may influence the rheological properties of the spinning solution as well as the structural characteristics of the resulting materials [10,11]. Consequently, PVA represents an attractive platform for the development of bio-based composite systems containing *Spirulina* and *Chlorella* biomass [9,10,11].

Electrospinning is one of the most versatile techniques for the fabrication of continuous nanofibers with diameters ranging from several micrometers down to a few tens of nanometers [10,11,12,13,14,15]. The resulting nanofibrous materials are characterized by high specific surface area, interconnected pore structure, and tunable morphology, which make them attractive for applications in filtration, tissue engineering, wound dressings, controlled release systems, and functional textiles [10,11,12,13,14,15]. Owing to its favorable rheological properties and excellent spinnability from aqueous solutions, PVA has become one of the most extensively investigated polymers for electrospinning [10,11]. The combination of PVA with bio-based additives therefore provides a promising route for the development of sustainable nanofibrous materials with tailored structural and transport properties [10,11,12,13,14,15].

The incorporation of microalgae and microalgae-derived compounds into electrospun materials has attracted increasing research interest in recent years [16,17,18,19]. Several studies have demonstrated that algal biomass can be successfully incorporated into polymer spinning solutions and subsequently processed in nanofibers, providing additional functionality through the presence of proteins, pigments, lipids, and antioxidant compounds [16,17,18,19,20]. Martins et al. [16] reported the electrospinning of PVA fibers containing bioactive pigments extracted from *Spirulina*, demonstrating the preservation of their functional properties after fiber formation. Similarly, microalgae-containing nanofibrous systems have been investigated as biohybrid materials, where living algal cells were immobilized within electrospun structures while maintaining biological activity [17,18]. Recent reviews have further highlighted the growing potential of algal nanofibers for biomedical, environmental, packaging, and advanced functional applications [16,17,18,19,20,21].

Despite these advances, the available literature remains largely focused on individual microalgal species and specific functional applications [16,19]. Most published studies investigate either *Spirulina*-based systems or *Chlorella*-based systems separately, while direct comparisons under identical electrospinning conditions are rarely reported [16,17,20,21]. As a result, the influence of algal species on solution properties, fiber formation, nanofiber morphology, and structural characteristics of the resulting nanomats remains insufficiently understood. This limitation makes it difficult to establish clear structure–property relationships and to determine how differences in the biochemical composition, cellular structure, and particle morphology of microalgae affect the performance of electrospun composite materials.

The incorporation of microalgal biomass therefore provides a sustainable approach for introducing naturally occurring functional compounds into electrospun PVA materials as an alternative to conventional synthetic functional additives. Besides improving the sustainability of the composite system, the incorporated biomass may modify the physicochemical properties of the spinning solution, influence fiber formation during electrospinning, and tailor the structural and transport properties of the resulting nanofibrous mats depending on the selected microalgal species.

Therefore, the aim of the present study is to develop electrospun PVA nanofibrous mats containing *Spirulina* and *Chlorella* biomass and to systematically evaluate how differences in the biochemical composition and morphology of *Spirulina* and *Chlorella* influence the physicochemical, structural, and transport properties of the resulting nanomats. Particular attention was paid to the relationship between solution characteristics, nanofiber morphology, nanomat growth behavior, surface porosity, and transport properties of textile-supported nanofibrous composites. In addition to the physicochemical characterization of the electrospun mats, the influence of the incorporated microalgae on air permeability and water-vapor transmission was investigated. The obtained results contribute to a better understanding of the role of microalgal biomass in electrospun PVA systems and provide useful information for the design of sustainable nanostructured materials for advanced filtration and functional textile applications.

## 2. Materials and Methods

### 2.1. Materials

Poly(vinyl alcohol) (PVA) supplied by Merck KGaA (Darmstadt, Germany), with a molecular weight of 72,000 g mol^−1^ and a degree of hydrolysis of 87–90%, was used as the polymer matrix. Commercial microalgal powders of *Spirulina* (*Arthrospira platensis*) and *Chlorella vulgaris* were purchased from Narayana Verlag GmbH (Kandern, Germany). According to the manufacturer’s specifications, the supplied *Spirulina* and *Chlorella* biomass powders had a particle size of 10–30 µm. Deionized water with an electrical conductivity of 1 μS cm^−1^ was used as the solvent.

### 2.2. Preparation of Electrospinning Solutions

The compositions of the electrospinning solutions used in this study, including the total solids content and the biomass powder-to-dry PVA ratio, are summarized in Table 1. A 9 wt% PVA solution was first prepared by dissolving the required amount of PVA in deionized water under continuous stirring at 760 rpm and heating at 80 °C for 30 min using a magnetic stirrer (MS7-H550-S, DLAB Scientific Co., Ltd., Beijing, China) until complete dissolution of the polymer and formation of a homogeneous solution. After cooling to room temperature, the PVA solution (200 mL) was divided into two equal portions (100 mL each). Then, 5 wt% of microalgal powder, calculated with respect to the mass of dry PVA, was added separately to each portion: *Spirulina* powder was added to one portion and *Chlorella* powder to the other. The resulting mixtures were stirred using a magnetic stirrer at 760 rpm for 30 min at room temperature to ensure homogeneous dispersion of the microalgal biomass in the electrospinning solutions. All electrospinning solutions were visually homogeneous, with no observable sedimentation or phase separation prior to the physicochemical characterization and electrospinning. The prepared electrospinning solutions were designated as PVA/*Spirulina* and PVA/*Chlorella*, respectively.

### 2.3. Electrospinning

**Figure 1:**
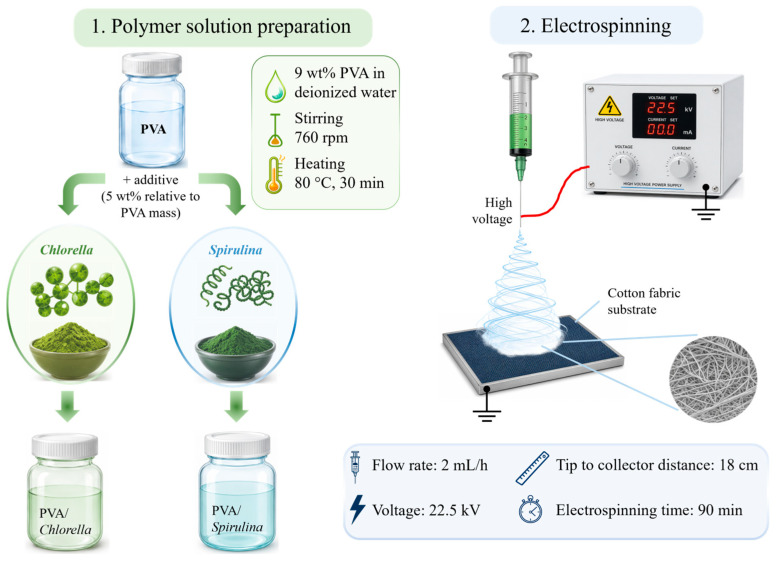
Schematic representation of the electrospinning process for PVA/microalgae composite nanofibers together with the laboratory electrospinning setup.

Electrospinning was performed using a single-nozzle laboratory setup consisting of a high-voltage power supply, a syringe pump (LEGATO 200, KD Scientific, Holliston, MA, USA), and a grounded collector, as presented in Figure 1. A neat PVA solution was electrospun under the same operating conditions and used as a reference for the SEM and FTIR analyses. The prepared PVA/*Spirulina* and PVA/*Chlorella* solutions were loaded into a plastic syringe equipped with a 27G blunt-tip needle (outer diameter 0.52 mm, length 1.9 cm). The polymer solution was delivered at a constant flow rate of 2 mL h^−1^ using a syringe pump. A voltage of 22.5 kV was applied between the needle and the collector, while the tip-to-collector distance was maintained at 18 cm.

Electrospinning was carried out for a total duration of 90 min under conditions ensuring stable Taylor cone at the needle tip and continuous jet deposition. The nanofibrous mat was characterized at selected time intervals during the deposition process, as described in the Results Section. Nanofibers were deposited directly onto a woven cotton denim fabric (100% cotton, thickness 0.7 mm, area density 345 g m^−2^) fixed onto the grounded collector and used as the substrate for nanofiber deposition. A total of 24 fabric–nanomat composite samples were prepared, comprising 12 PVA/*Spirulina* and 12 PVA/*Chlorella* specimens distributed among the investigated electrospinning time points. A schematic illustration of the preparation procedure and electrospinning process is presented in Figure 1.

A woven cotton fabric was selected as the collecting substrate instead of a conventional metallic collector because the objective of this study was to investigate textile-supported nanofibrous composites. Our previous studies demonstrated that the properties of the textile substrate, particularly its hydrophilicity and surface characteristics, significantly influence nanofiber deposition, nanomat growth, and the morphology of the resulting textile-supported composites. Therefore, electrospinning was performed directly onto the woven cotton fabric to obtain integrated fabric–nanomat composites suitable for subsequent structural and transport characterization [22,23].

### 2.4. Characterization

#### 2.4.1. Physicochemical Characterization of the Electrospinning Solutions

The electrical conductivity of the electrospinning solutions was measured using a conductivity meter (CM-40G, TOA Electronics Ltd., Aichi, Japan). The viscosity was determined at room temperature using a rotational viscometer (Fungilab Smart L, Fungilab S.A., Barcelona, Spain) equipped with spindle No. 63 operating at 100 rpm. Each value was recorded after stabilization of the instrument reading. Surface tension measurements were performed using the pendant-drop method. For each electrospinning solution, electrical conductivity, viscosity, and surface tension were measured in five independent replicates, and the results are reported as mean ± standard deviation (SD).

#### 2.4.2. Scanning Electron Microscopy (SEM)

Small pieces of the electrospun nanomats were mounted on aluminum stubs using conductive carbon tape and sputter-coated with a Pd/Pt layer prior to observation. The morphology of the electrospun nanofibers was examined using a scanning electron microscope (SEM, VE-8800, Keyence Co., Tokyo, Japan). The average fiber diameter and diameter distribution were determined from SEM micrographs using image analysis software ImageJ (version 1.54g, National Institutes of Health, Bethesda, MD, USA). Fiber diameter analysis was performed using five representative SEM micrographs, and a total of 300 fibers were measured for each sample to determine the average fiber diameter and diameter distribution. Surface porosity was determined from five representative SEM images and is reported as mean ± standard deviation (SD).

#### 2.4.3. Fourier Transform Infrared Spectroscopy (FTIR)

The electrospun nanomat samples were analyzed by Fourier-transform infrared spectroscopy (FTIR) using a Varian 3100 FT-IR Excalibur Series spectrophotometer to identify the functional groups and evaluate the interactions between PVA and the incorporated microalgal biomass.

#### 2.4.4. Nanomat Thickness and Growth Characterization

The thickness and projected surface area of the deposited nanofibrous mats were measured using a laser scanning microscope (VK-9710K, Keyence Co., Tokyo, Japan). The nanomat thickness was determined from cross-sectional height profiles obtained from the laser microscope, while the projected nanomat area was calculated from the recorded top-view images using the instrument software.

The mass increase in the deposited nanomat during electrospinning was determined gravimetrically by weighing the collector together with the deposited nanomat at predetermined electrospinning time intervals using an analytical balance BAS 31 Plus (Boeco, Hamburg, Germany). The mass of the deposited nanomat was calculated from the difference between the measured mass after deposition and the initial mass of the collector.

#### 2.4.5. Air Permeability

Air permeability measurements were performed on the fabric–nanomat composite specimens using an FX 3300 LabAir III instrument (Textest AG, Schwerzenbach, Switzerland).

#### 2.4.6. Water Vapor Permeability

Water-vapor permeability and thermal transport properties were determined using a Permetest 100 instrument (Sensora Instruments, Liberec, Czech Republic) according to ISO 11092 [24]. Prior to testing, all fabric–nanomat composite specimens were conditioned for 24 h at 21 °C and 64% relative humidity.

#### 2.4.7. Droplet Profile and Interfacial Tension Measurements

The interfacial behavior of the electrospinning solutions on the denim substrate was evaluated by droplet-profile analysis using an automatic contact angle measuring instrument (LR-SDC-350, Lonroy, Dongguan, China). A 5 μL droplet of the corresponding electrospinning solution (PVA, PVA/*Spirulina*, or PVA/*Chlorella*) was carefully dispensed onto the denim fabric surface through a stainless-steel needle with an outer diameter of 520 μm. The droplet profile was recorded, and the corresponding interfacial tension was calculated using the instrument software. Measurements were performed at 18.4 °C, and at least five independent measurements were carried out for each electrospinning solution.

## 3. Results and Discussion

### 3.1. Physicochemical Properties of the Electrospinning Solutions

The rheological and physicochemical properties of the electrospinning solutions significantly affect jet stability and the morphology of the resulting nanofibers. The measured viscosity, electrical conductivity, and surface tension values are presented in Table 2.

As shown in Table 2, the addition of *Spirulina* and *Chlorella* biomass increased the viscosity, electrical conductivity, and surface tension of the PVA solution. The increase can be attributed to the presence of proteins, polysaccharides, pigments, minerals, and other polar compounds in the algal biomass, which interact with the hydroxyl groups of PVA through hydrogen bonding [9,11]. In addition, the dispersed microalgal powder particles may also contribute to the increased viscosity by increasing the effective solid volume fraction of the electrospinning solutions, thereby restricting polymer chain mobility. These interactions restrict molecular mobility and modify the rheological behavior of the solutions. The PVA/*Chlorella* solution exhibited slightly higher viscosity and electrical conductivity than the PVA/*Spirulina* system, indicating differences in the chemical composition and dispersion behavior of the two microalgal species.

The observed changes in the physicochemical properties of the composite electrospinning solutions relative to the neat PVA solution are also influenced by the different biochemical compositions of *Spirulina* and *Chlorella*. The presence of proteins, lipids, carbohydrates, and mineral components introduces additional functional groups capable of interacting with the PVA macromolecules. Since electrospinning was performed from aqueous solutions, both intermolecular and intramolecular hydrogen-bond interactions are expected between the hydroxyl groups of PVA and the functional groups present in the microalgal biomass. These interactions contribute to the observed increases in viscosity and surface tension.

Besides their different biochemical compositions, the two microalgae also exhibit markedly different particle morphologies, which are expected to influence their behavior during electrospinning. *Chlorella vulgaris* consists of nearly spherical unicellular particles, whereas *Spirulina* (*Arthrospira platensis*) forms filamentous spiral–helical trichomes. These differences in particle geometry are likely to affect biomass dispersion within the PVA electrospinning solution, particle–polymer interactions, and the hydrodynamic behavior of the dispersed biomass under the elongational flow generated during electrospinning. Consequently, the intrinsic morphology of the microalgal particles may contribute to the observed differences in solution properties, nanofiber formation, nanomat growth, and the transport characteristics of the resulting composite nanomats.

**Figure 2:**
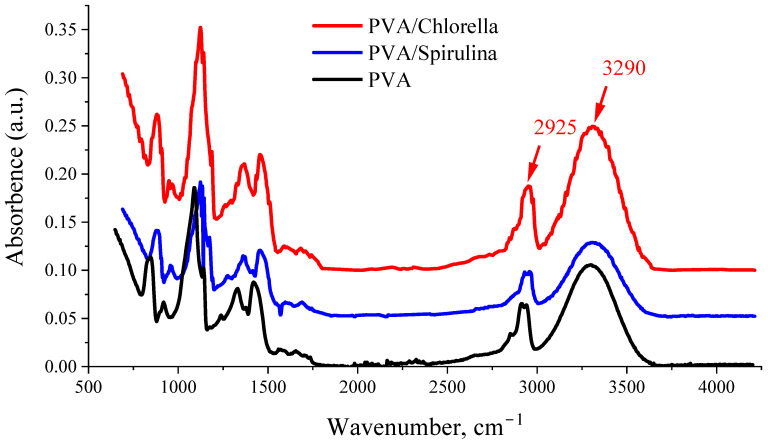
FTIR spectra of neat PVA and electrospun PVA/*Spirulina* and PVA/*Chlorella* composite nanomats. The spectra demonstrate that the characteristic absorption bands of PVA are retained after incorporation of the microalgal biomass.

The observed changes in viscosity, electrical conductivity, and surface tension suggest that both the biochemical composition and the intrinsic morphology of the incorporated microalgal biomass influence its interactions with the PVA matrix. To gain further insight into the chemical structure of the resulting composite nanofibers and to identify the functional groups associated with the incorporated microalgal biomass, FTIR spectroscopy was performed. The corresponding spectra are presented in Figure 2.

Figure 2 presents the FTIR spectra of neat PVA together with the electrospun PVA/*Spirulina* and PVA/*Chlorella* composite nanomats. The FTIR spectrum of neat PVA exhibits the characteristic absorption bands reported in the literature, including a broad absorption band centered at approximately 3290 cm^−1^ assigned to O–H stretching vibrations associated with intra- and intermolecular hydrogen bonding, and a band at approximately 2925 cm^−1^ corresponding to C–H stretching vibrations. Additional characteristic bands at 1141, 1088, 916 and 822 cm^−1^ are associated with C–O stretching, CH_2_ rocking and C–C stretching vibrations of PVA [25].

The FTIR spectra of the electrospun PVA/*Spirulina* and PVA/*Chlorella* composite nanomats largely retain the characteristic absorption bands of neat PVA, indicating that the polymer matrix remains the dominant structural component after incorporation of the microalgal biomass. The broad absorption band centered at approximately 3290 cm^−1^ is primarily attributed to O–H stretching vibrations of PVA. Following the incorporation of the microalgal biomass, this region may also include overlapping contributions from N–H stretching vibrations originating from proteins present in *Spirulina* and *Chlorella*. However, owing to the broad and intense O–H absorption of PVA, the individual contributions of these functional groups cannot be unambiguously resolved [25,26]. The characteristic C–H stretching band at approximately 2925 cm^−1^ remains clearly visible in all spectra, indicating that the incorporation of the microalgal biomass does not alter the principal chemical structure of the PVA matrix. Minor spectral differences are observed in the 1500–1800 cm^−1^ region, although these features cannot be assigned unequivocally because of the overlap with the characteristic absorption bands of PVA. A weak spectral feature observed near 1735 cm^−1^ in the PVA/*Chlorella* spectrum may be associated with the C=O stretching vibrations of ester groups originating from lipid and fatty-acid components of the incorporated microalgal biomass. Owing to its low intensity, this assignment should be regarded as tentative. The weak band observed in the 822–825 cm^−1^ region overlaps with a characteristic PVA absorption and may additionally contain contributions from nucleotide-ring and chlorophyll-related vibrations reported for algal biomass [26]; therefore, its assignment is tentative. The light-green coloration of the electrospun PVA/*Chlorella* nanofibrous mats further supports the presence and retention of chlorophyll pigments after the electrospinning process.

The close similarity of the spectra provides no clear evidence of the formation of new covalent bonds during solution preparation and electrospinning. Instead, the interactions between PVA and the incorporated microalgal biomass are predominantly physical and are mainly governed by hydrogen bonding. These interactions facilitate the homogeneous incorporation of the microalgal powder into the PVA matrix and are consistent with the changes in viscosity, electrical conductivity, and surface tension observed for the electrospinning solutions (Table 2).

### 3.2. Wetting Behavior of Electrospinning Solutions on the Textile Substrate

**Figure 3:**
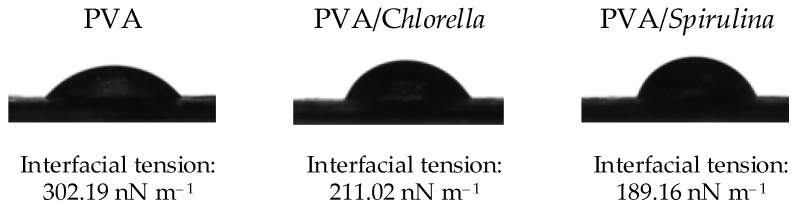
Representative droplet profiles of PVA, PVA/*Chlorella* and PVA/*Spirulina* electrospinning solutions deposited on the denim substrate and the corresponding interfacial tension values.

The wetting behavior of the electrospinning solutions on the denim substrate was evaluated by droplet profile analysis and interfacial tension measurements. Representative droplet profiles together with the corresponding interfacial tension values are presented in Figure 3.

The reference PVA solution exhibited the highest interfacial tension value (302.19 nN m^–1^), whereas lower values were obtained for the electrospinning solutions containing microalgal biomass. The interfacial tension decreased to 211.02 nN m^–1^ for the PVA/*Chlorella* solution and to 189.16 nN m^–1^ for the PVA/*Spirulina* solution.

The observed reduction in interfacial tension can be attributed to the presence of proteins, lipids, polysaccharides, and other surface-active compounds originating from the microalgal biomass. The stronger effect observed for the PVA/*Spirulina* solution suggests a greater contribution of surface-active constituents and more pronounced spreading on the denim substrate compared with the PVA/*Chlorella* solution.

The observed differences in wetting behavior may influence the spreading and penetration of the electrospinning solutions on the textile substrate during nanomat formation. Consequently, these differences may indirectly affect the morphology and structural development of the deposited nanofibrous layers.

### 3.3. Morphology and Diameter Distribution of Electrospun Nanofibers

**Figure 4:**
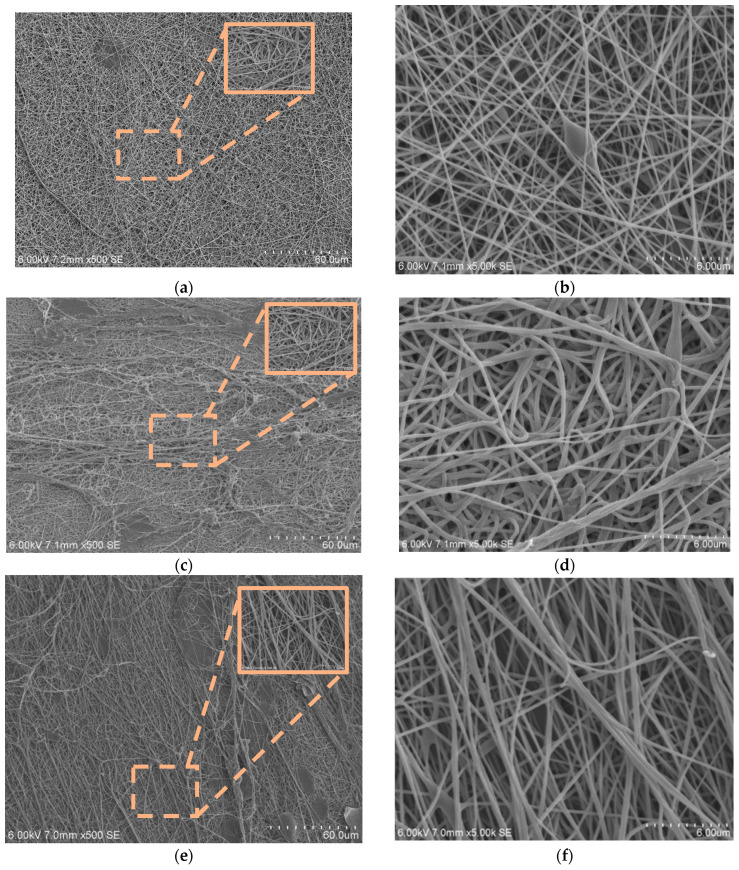
SEM micrographs of electrospun nanofibrous mats prepared from (**a**,**b**) neat PVA, (**c**,**d**) PVA/*Spirulina*, and (**e**,**f**) PVA/*Chlorella*. Panels (**a**,**c**,**e**) show the overall morphology of the electrospun nanofibrous mats, while panels (**b**,**d**,**f**) present higher-magnification images of the regions highlighted in the corresponding overview micrographs. Scale bars: 60 μm in (**a**,**c**,**e**) and 6 μm in (**b**,**d**,**f**).

**Figure 5:**
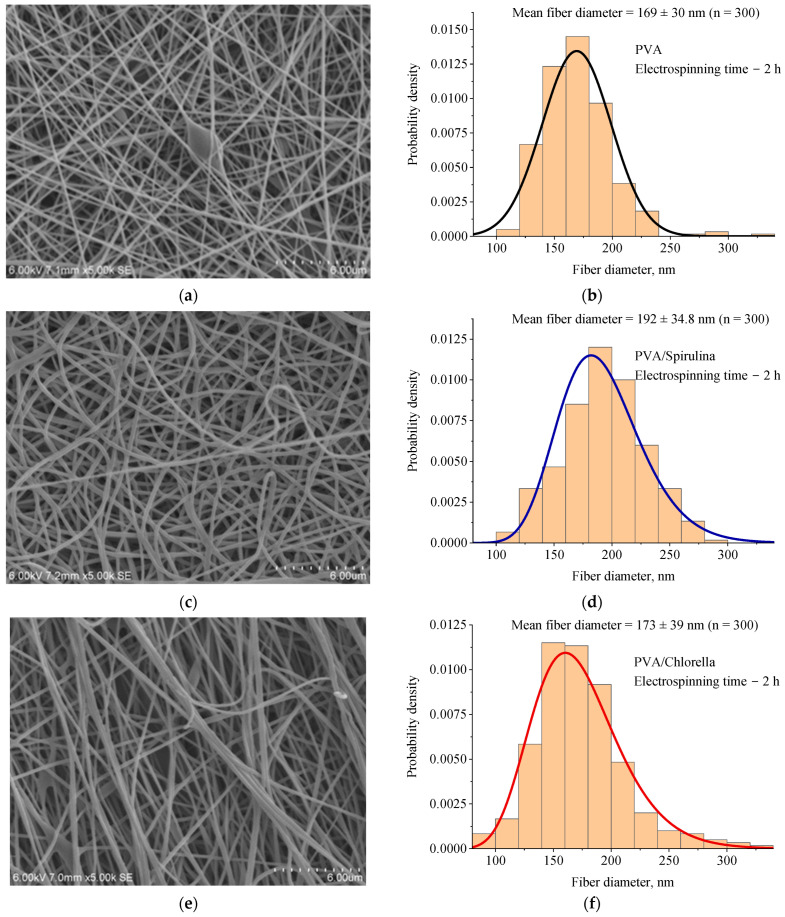
Representative SEM micrographs and corresponding fiber diameter distributions of electrospun nanofibers: (**a**) SEM image of neat PVA nanofibers; (**b**) fiber diameter distribution of neat PVA nanofibers; (**c**) SEM image of PVA/*Spirulina* nanofibers; (**d**) fiber diameter distribution of PVA/*Spirulina* nanofibers; (**e**) SEM image of PVA/*Chlorella* nanofibers; (**f**) fiber diameter distribution of PVA/*Chlorella* nanofibers.

The morphology of the electrospun nanofibers was examined using scanning electron microscopy (SEM). Representative SEM micrographs of neat PVA together with PVA/*Spirulina* and PVA/*Chlorella* electrospun nanofibrous mats are presented in Figure 4 and Figure 5. The inclusion of neat PVA enables a direct comparison of the influence of the incorporated microalgal biomass on fiber morphology and diameter.

The incorporation of *Spirulina* and *Chlorella* biomass did not alter the ability of the solutions to form continuous nanofibers. In both cases, continuous fibrous structures without visible bead defects or pronounced surface irregularities at the SEM magnification used were obtained. Minor differences in the visual appearance of the nanofibrous network and fiber diameter distribution were observed depending on the type of incorporated microalgal biomass. These changes can be attributed to the increased viscosity and electrical conductivity of the electrospinning solutions (Table 2), which affect jet stretching and solvent evaporation during fiber formation.

Besides the changes in viscosity and electrical conductivity, the differences in the intrinsic morphology of the incorporated microalgal biomass may also have contributed to the observed differences in fiber morphology and diameter.

As shown in Figure 4, both microalgal species enabled the formation of continuous, bead-free nanofibrous structures without visible surface irregularities at the SEM magnification used. No distinct biomass particles were visible on the fiber surfaces at the applied SEM magnification.

The bead-free morphology observed for both composite systems indicates that the electrospinning process remained stable after the incorporation of microalgal biomass. Excessively rapid solvent evaporation may lead to bead formation and increased fiber diameter, whereas slower solvent removal facilitates jet stretching and polymer-chain alignment, resulting in more uniform fiber morphology [27,28]. The interconnected nanofibrous networks observed in both samples further confirm that neither *Spirulina* nor *Chlorella* adversely affected fiber formation under the applied electrospinning conditions.

The mean fiber diameter of neat PVA was 169 ± 30 nm. Following the incorporation of microalgal biomass, the mean fiber diameter increased to 192 ± 34 nm for PVA/*Spirulina* and to 173 ± 39 nm for PVA/*Chlorella* (Figure 5). Thus, *Spirulina* produced a more pronounced increase in fiber diameter, whereas the mean diameter of the PVA/*Chlorella* fibers remained close to that of neat PVA.

Although the PVA/*Chlorella* solution exhibited a higher viscosity (Table 2), slightly thinner fibers were obtained. This suggests that, in addition to viscosity, other parameters such as electrical conductivity and molecular interactions between PVA and the microalgal components also influenced jet stretching and fiber formation during electrospinning.

The fiber diameter distributions were determined from measurements of 300 individual fibers for each sample. The three histograms exhibited unimodal diameter distributions with a slight tail toward larger fiber diameters, indicating stable fiber formation and relatively uniform fiber formation. The measured standard deviations indicate moderate variation in fiber diameter within each sample. The measured standard deviations (±34 nm for PVA/*Spirulina* and ±39 nm for PVA/*Chlorella*) indicate a relatively limited variation in fiber diameter within each sample and were used as descriptive statistical parameters for comparison of the two composite systems.

### 3.4. Growth Kinetics of the Electrospun Nanomat

**Figure 6:**
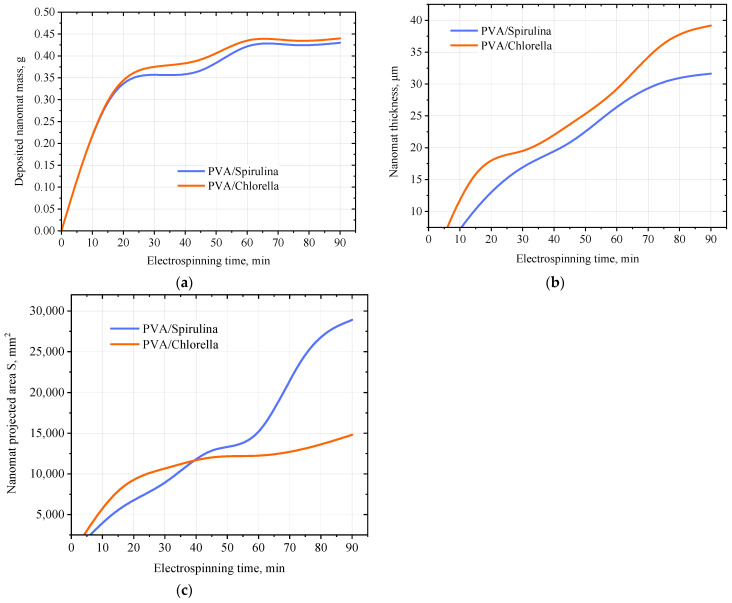
Evolution of the deposited (**a**) nanofiber mass, (**b**) nanomat thickness, and (**c**) projected area as a function of electrospinning time.

The growth kinetics of nanomat formation during electrospinning were investigated by monitoring time-dependent changes in deposited mass, nanomat thickness, and projected nanomat area. The deposited mass was determined gravimetrically, whereas nanomat thickness and projected area were measured by laser scanning microscopy. The corresponding kinetic curves for the PVA/*Spirulina* and PVA/*Chlorella* systems are presented in Figure 6, while the parameters of the piecewise linear regression analysis are summarized in Table 3.

The mass accumulation kinetics were very similar for both systems. The initial mass accumulation rate of PVA/*Spirulina* was approximately 1.12 times higher than that of PVA/*Chlorella*. In both systems, the growth rate decreased by a factor of approximately 16 during the second kinetic regime, indicating a similar transition to a substantially slower accumulation regime.

More pronounced differences were observed for nanomat thickness. The initial thickness growth rate of PVA/*Chlorella* was approximately 1.49 times higher than that of PVA/*Spirulina*. Following the transition to the second growth regime, the thickness growth rate decreased by factors of 2.58 and 3.06 for PVA/*Spirulina* and PVA/*Chlorella*, respectively, suggesting a gradual reduction in vertical growth as the deposition process progressed.

The most pronounced differences were observed in the evolution of the nanomat area. During the initial stage, the area expansion rate of PVA/*Chlorella* was nearly two times higher than that of PVA/*Spirulina*. However, the subsequent behavior differed substantially. While the area growth rate of PVA/*Chlorella* decreased by a factor of approximately 9.2, the PVA/*Spirulina* system exhibited a 1.56-fold increase in the area expansion rate during the second regime. Consequently, the projected area growth rate of PVA/*Spirulina* became approximately 7.25 times higher than that of PVA/*Chlorella* at longer deposition times.

The present investigation was primarily intended to provide a proof-of-concept comparison of two commercially available microalgal biomasses under identical electrospinning conditions. Understanding the relationship between the characteristics of the incorporated biomass and the resulting nanomat growth behavior may facilitate the rational design of bio-based electrospun materials with tailored structural and transport properties.

### 3.5. Surface Porosity of the Electrospun Nanomats

The surface porosity of the electrospun PVA/*Spirulina* and PVA/*Chlorella* nanomats was evaluated from SEM micrographs using ImageJ software. Prior to analysis, the images were converted to 8-bit grayscale, the scale bar was removed, and the background was corrected.

**Figure 7:**
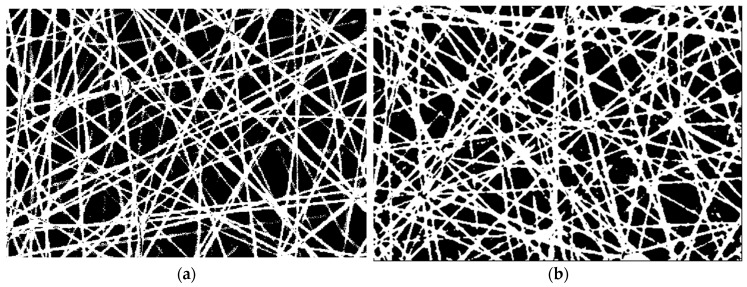
Representative binarized SEM images used for the determination of surface porosity of electrospun nanomats: (**a**) PVA/*Spirulina* and (**b**) PVA/*Chlorella*. Surface porosity was calculated from the pore area fraction obtained after image binarization using ImageJ software.

Representative binarized SEM images used for the porosity analysis are presented in Figure 7. The pore area fraction was determined after image binarization and calculated as the ratio of the pore area to the total analyzed area. At least five representative SEM images were analyzed for each sample, and the results were expressed as mean ± standard deviation (SD).

**Figure 8:**
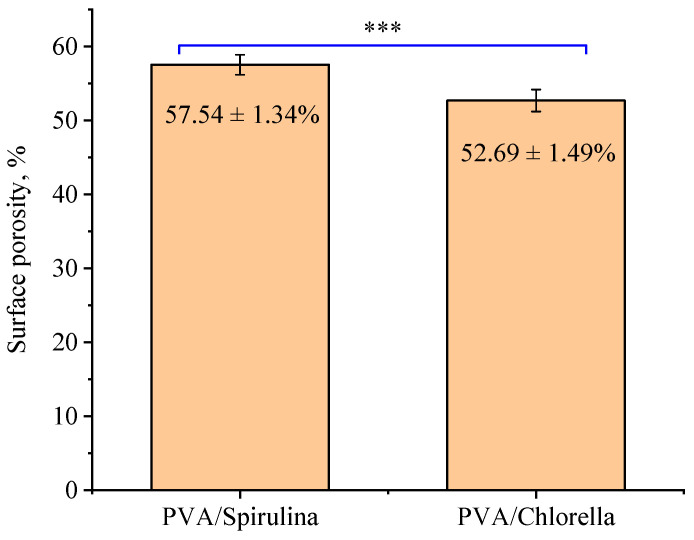
Comparison of the surface porosity of electrospun PVA/*Spirulina* (57.54 ± 1.34%) and PVA/*Chlorella* (52.69 ± 1.49%) nanomats. Error bars represent standard deviation (n = 5). Statistical significance: *** *p* < 0.001.

The calculated surface porosity values are summarized in Table 4 and graphically presented in Figure 8. PVA/*Spirulina* nanomats exhibited significantly higher surface porosity (57.54 ± 1.34%) than PVA/*Chlorella* nanomats (52.69 ± 1.49%), as confirmed by Welch’s *t*-test (*p* < 0.001).

The higher surface porosity of the PVA/*Spirulina* nanomats is consistent with the binarized SEM images shown in Figure 7, which indicate a more open fibrous network, and is quantitatively confirmed by the results presented in Figure 8. In contrast, the PVA/*Chlorella* nanomats exhibited a denser fiber arrangement and a lower fraction of open pores. These structural differences may be associated with the distinct physicochemical properties of the electrospinning solutions (Table 2), particularly their viscosity and electrical conductivity, which influence fiber deposition and packing during nanomat formation. The higher surface porosity of the PVA/*Spirulina* nanomats may be advantageous for applications requiring enhanced mass transport, fluid exchange, or accessible surface area.

### 3.6. Through-Plane Transport Properties of the Fabric–Nanomat Composites

To evaluate how the structural differences between the electrospun nanomats affect the transport behavior of the resulting fabric–nanomat composites, air permeability and relative water vapor permeability were investigated.

#### 3.6.1. Air Permeability of the Fabric–Nanomat Composites

Air permeability is an important transport property of textile materials and reflects the ability of air to pass through a porous structure under a defined pressure difference [29,30]. In the present study, the influence of electrospinning time and microalgal biomass type on the air permeability of the fabric–nanomat composites was investigated.

**Figure 9:**
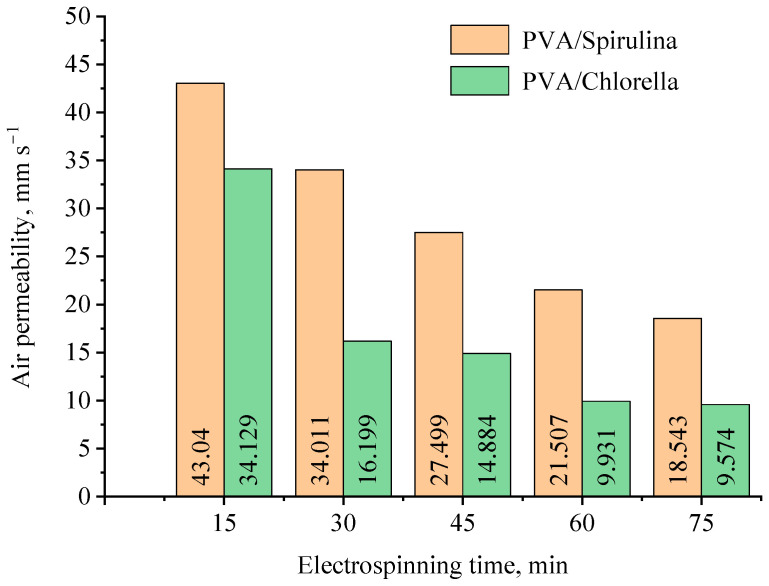
Air permeability of the fabric–nanomat composites as a function of electrospinning time.

The results presented in Figure 9 show a gradual decrease in air permeability with increasing electrospinning time for both PVA/*Spirulina* and PVA/*Chlorella* systems. This behavior is associated with the continuous accumulation of nanofibers on the textile substrate, resulting in an increase in nanomat thickness (Figure 6b) and a reduction in the effective pore size available for airflow.

At all electrospinning times, the composites containing PVA/*Chlorella* nanomats exhibited lower air permeability than the corresponding PVA/*Spirulina* composites (Figure 9). The difference became progressively more pronounced with increasing deposition time. After 75 min of electrospinning, the air permeability decreased to 9.54 mm s^−1^ for PVA/*Chlorella* and 18.54 mm s^−1^ for PVA/*Spirulina*. This behavior is consistent with the greater nanomat thickness obtained for PVA/*Chlorella* (Figure 6b) and its lower surface porosity (52.69 ± 1.49%) compared to PVA/*Spirulina* (57.54 ± 1.34%) (Table 4).

The SEM observations (Figure 4 and Figure 5) further support these findings, showing a more compact fibrous network in the PVA/*Chlorella* samples. Consequently, the PVA/*Chlorella* nanomat acts as a more effective barrier to airflow, whereas the more porous PVA/*Spirulina* nanomat preserves a higher degree of air permeability. These results demonstrate that the type of incorporated microalgal biomass influences not only the morphology and porosity of the electrospun nanomats but also the transport properties of the resulting fabric–nanomat composites.

#### 3.6.2. Relative Water Vapor Permeability of the Fabric–Nanomat Composites

**Figure 10:**
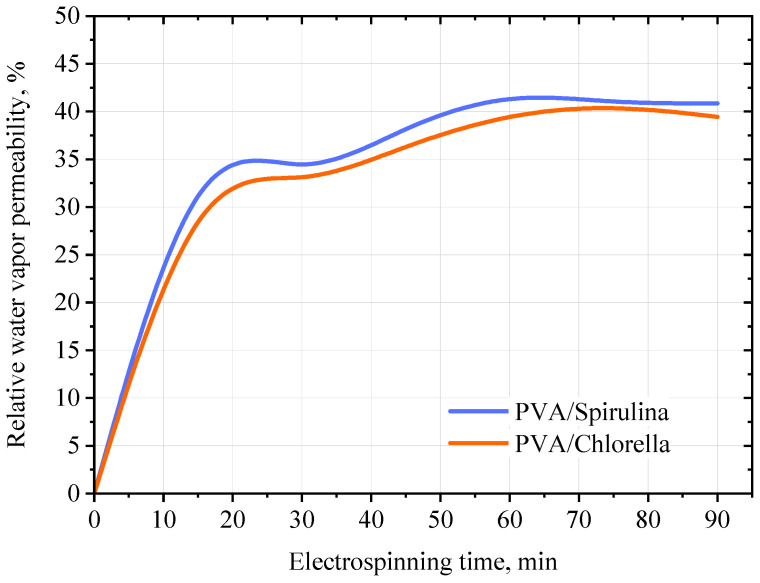
Relative water vapor permeability of the fabric–nanomat composites as a function of electrospinning time.

Water vapor transport is one of the key parameters governing the physiological comfort of textile materials and fibrous structures intended for biomedical and wearable applications [10,11,31]. The influence of electrospinning time on the relative water vapor permeability of fabric–nanomat composites containing PVA/*Spirulina* and PVA/*Chlorella* nanomats is presented in Figure 10.

For both systems, the relative water vapor permeability increased rapidly during the initial stage of electrospinning and gradually approached a plateau after approximately 60–70 min. The permeability increased from nearly zero to approximately 33–34% within the first 20 min of electrospinning, followed by a slower increase until reaching final values of about 40–41%. This behavior reflects the progressive formation of a porous nanofibrous layer capable of facilitating moisture transport through the composite structure.

Only minor differences were observed between the two types of microalgae-containing nanomats. Throughout most of the investigated time interval, the PVA/*Spirulina* composites exhibited slightly higher relative water vapor permeability than the corresponding PVA/*Chlorella* composites. However, after prolonged electrospinning (75–90 min), both systems reached similar permeability values, indicating comparable water vapor transport characteristics.

The observed behavior differs from that obtained for air permeability (Figure 9), where markedly lower values were measured for the PVA/*Chlorella* composites. Similarly, the surface porosity analysis revealed statistically significant differences between the two nanomats (Table 4 and Figure 8). Despite these structural differences, the relative water vapor permeability remained nearly unchanged. This suggests that both nanofibrous structures provide sufficiently interconnected pathways for moisture diffusion through the composite, even though their airflow resistance and pore architecture differ.

The results demonstrate that the incorporation of either *Spirulina* or *Chlorella* biomass does not adversely affect the moisture transport capability of the resulting fabric–nanomat composites. The preservation of high relative water vapor permeability, together with the reduced air permeability achieved after nanomat deposition, is advantageous for applications requiring a balance between barrier performance and wearer comfort.

## 4. Conclusions

Electrospun nanofibrous mats based on poly(vinyl alcohol) (PVA) containing two types of microalgal biomass, *Spirulina* and *Chlorella*, were successfully prepared and characterized. The influence of the microalgal species on the physicochemical properties of the electrospinning solutions, nanofiber morphology, nanomat growth behavior, surface porosity, and transport properties of fabric–nanomat composites was systematically investigated.

The incorporation of both types of microalgal biomass increased the viscosity, electrical conductivity, and surface tension of the PVA solutions, indicating interactions between the algal biomass and the polymer matrix. FTIR analysis showed that the composite nanomats retained the characteristic absorption bands of neat PVA, with minor spectral features consistent with the incorporated microalgal biomass. The spectra did not indicate the formation of new covalent bonds.

SEM observations revealed the formation of continuous, bead-free nanofibers without visible surface irregularities at the SEM magnification used. The average fiber diameters were 192 ± 34 nm for PVA/*Spirulina* and 173 ± 39 nm for PVA/*Chlorella*, demonstrating that both biomass-containing PVA solutions could be successfully electrospun without compromising nanofiber formation.

The type of incorporated microalgae affected the growth kinetics and structural development of the electrospun nanomats. While similar mass accumulation behavior was observed for both systems, more pronounced differences were detected in nanomat thickness and area evolution during electrospinning. The PVA/*Chlorella* system produced thicker nanomats, whereas the PVA/*Spirulina* system exhibited greater projected-area expansion at longer deposition times.

Surface porosity analysis demonstrated significantly higher porosity for PVA/*Spirulina* nanomats (57.54 ± 1.34%) compared with PVA/*Chlorella* nanomats (52.69 ± 1.49%). These structural differences were reflected in the transport properties of the fabric–nanomat composites. PVA/*Spirulina* composites maintained higher air permeability, whereas PVA/*Chlorella* composites exhibited greater resistance to airflow due to their denser structure. In contrast, both systems showed comparable relative water vapor permeability, indicating that structural differences between the nanomats had a limited effect on moisture transport.

Overall, the results demonstrate that the choice of microalgal species influences the morphology, porosity, and transport properties of electrospun PVA nanomaterials. Among the investigated systems, PVA/*Spirulina* exhibited a more porous structure and higher air permeability, whereas PVA/*Chlorella* formed denser nanomats with lower air permeability and greater thickness. The obtained fabric–nanomat composites combine good vapor transport characteristics with tunable air permeability, demonstrating their potential for further development as functional textile and filtration-related materials.

## Figures and Tables

**Table 1:** Composition of the electrospinning solutions, including total solids content and biomass content relative to dry PVA.

Sample	Total Solids Content, wt%	Biomass Content (wt% Relative to Dry PVA)
PVA	9.00	0
PVA/*Spirulina*	9.45	5
PVA/*Chlorella*	9.45	5

**Table 2:** Physicochemical properties of the electrospinning solutions used for the preparation of PVA/*Spirulina* and PVA/*Chlorella* nanofibers.

Solution Type	Viscosity, Pa.s	Electrical Conductivity, µS cm^−1^	Surface Tension, mN m^−1^
PVA	0.191 ± 0.0012	0.29 ± 0.007	40.3 ± 0.1
PVA/*Spirulina*	0.283 ± 0.0013	0.69 ± 0.010	61.8 ± 0.1
PVA/*Chlorella*	0.316 ± 0.0010	0.76 ± 0.010	66.3 ± 0.1

Values are presented as mean ± standard deviation (SD) based on five independent measurements (n = 5).

**Table 3:** Summary of the piecewise linear regression parameters describing the time-dependent evolution of mass accumulation (m), deposited nanomat thickness (d), and projected nanomat area (S) in PVA/*Spirulina* and PVA/*Chlorella* systems. The values are presented as regression coefficients ± standard error (SE), while R^2^ denotes the coefficient of determination.

Parameter	Solution	Region	Time Interval, min	Regression Equation	Slope ± SE (Standard Error)	Intercept ± SE (Standard Error)	R^2^
**Deposited nanomat mass *m*, g**	PVA/*Spirulina*	I	0–15.5	m = 0.02279t + 0.00223	0.0228 ± 0.0003	0.00223 ± 0.00293	0.999
		II	15.5–90.0	m = 0.00142t + 0.31701	0.00142 ± 0.00016	0.31701 ± 0.00932	0.782
	PVA/*Chlorella*	I	0–18.6	m = 0.02041t + 0.01458	0.02041 ± 0.00141	0.01458 ± 0.01576	0.988
		II	18.6–90.0	m = 0.00126t + 0.34107	0.00126 ± 0.00014	0.34107 ± 0.00829	0.796
**Nanomat thickness** ***d*** **, µm**	PVA/*Spirulina*	I	0–15.5	d = 0.7327t + 0.03045	0.73266 ± 0.00423	0.0305 ± 0.0397	0.999
		II	15.5–90.0	d = 0.2838t + 8.43558	0.28381± 0.00970	8.4356 ± 0.5663	0.975
	PVA/*Chlorella*	I	0–18.6	d = 1.0939t + 0.89564	1.09397 ± 0.08649	0.8956 ± 0.9678	0.970
		II	18.6–90.0	d = 0.3572t + 8.41866	0.35724 ± 0.01260	8.4187 ± 0.7502	0.975
**projected nanomat area *S*, mm^2^**	PVA/*Spirulina*	I	0–40.3	S = 272.82t + 980.81	272.8234 ± 14	980.8066 ± 324	0.971
		II	40.3–90.0	S = 425.46t − 8318.85	425.4623 ± 39.9795	−8318.8520 ± 2728.2235	0.890
	PVA/*Chlorella*	I	0–18.6	S = 540.92t + 353.27	540.9223 ± 34.1111	353.2675 ± 381.6907	0.981
		II	18.6–90.0	S = 58.70t + 8962.47	58.6953 ± 4.0014	8962.4654 ± 238.2191	0.911

Values in the “Slope ± SE” column are presented in g min^−1^ for deposited mass, µm min^−1^ for nanomat thickness, and mm^2^ min^−1^ for nanomat projected area. SE denotes the standard error of the regression coefficient.

**Table 4:** Comparison of the surface porosity of electrospun PVA/*Spirulina* and PVA/*Chlorella* fibrous mats.

**Sample**	**Surface Porosity, %**	$Surface\;porosity=\frac{A_{pores}}{A_{total}}\times 100$
PVA/*Spirulina*	57.54 ± 1.34	
PVA/*Chlorella*	52.69 ± 1.49	

## Data Availability

All the data and information relevant to the article are present within the article itself.
